# Anstie on Epidemics

**Published:** 1866-12

**Authors:** 


*Anstie on Epidemics.*—This work of ninety-five pages, from the press of Lippincott & Co., Philadelphia, has been sent us by the publishers. We have not had time to peruse it. We shall do so, however, and give our opinion of it.

It is claimed tor Dr. Anstie, that he is distinguished for his scientific attainments, and he has doubtless presented a popular treatise of much merit; but we differ with the publishers as to the idea of popularizing medicine, or anything else. Popular literature is not likely to improve either the taste or morals of a people. Popular constitutions and laws soon react upon the public mind, and the inate respect of the people for the law-making, and law-administering power degenerates into contempt for both. Might this not be true in medicine also. We believe that much might be accomplished in this direction, by shortening, and anglicizing many of the long hard terms in which the science of medicine is enwrapt, and to this extent we are with the publishers.

